# On the degradation mechanisms of quantum-dot light-emitting diodes

**DOI:** 10.1038/s41467-019-08749-2

**Published:** 2019-02-15

**Authors:** Song Chen, Weiran Cao, Taili Liu, Sai-Wing Tsang, Yixing Yang, Xiaolin Yan, Lei Qian

**Affiliations:** 1 College of Chemistry, Chemical Engineering and Materials Science, Soochow University, 199 Ren’ai Road, Suzhou Industrial Park, Suzhou, 215123 Jiangsu China; 2 TCL Corporate Research, 1001 Zhongshan Park Road, Nanshan District, Shenzhen, 518067 Guangdong China; 3 Department of Materials Science and Engineering, City University of Hong Kong, 83 Tat Chee Avenue, Kowloon Tong, Hong Kong SAR China

## Abstract

The operating lifetime of blue quantum-dot light-emitting diodes (QLED) is currently a short slab for this emerging display technology. To pinpoint the origin of device degradation, here we apply multiple techniques to monitor the electric-field distribution and space-charge accumulation across the multilayered structure before and after lifetime tests. Evident by charge-modulated electro-absorption and capacitance-voltage characteristics, the excited electrons in blue quantum dots (QD) are prone to cross the type II junction between the QD emission layer and the electron-transporting layer (ETL) due to the offset of conduction band minimum, leading to space-charge accumulation and operating-voltage rise in the ETL. Therefore, unlike those very stable red devices, of which the lifetime is primarily limited by the slow degradation of hole-transporting layer, the poor lifetime of blue QLED originates from the fast degradation at the QD-ETL junction. Materials engineering for efficient electron injection is prerequisite for the boost of operating lifetime.

## Abstract (web summary)

Wide application of quantum dot light emitting diodes (QLED) in display technology is hindered by the poor lifetime of the blue QLEDs. Here, the degradation mechanism is shown to originate from space charge accumulation in the electron-transporting layer enabling improvements in blue QLED lifetimes.

## Introduction

Combining the features of light-emitting quantum dots and solution processing, QLED has become an emerging display technology potentially capable of 100% Rec.2020 color gamut, high luminance efficiency and low-cost manufacturing^1–3^. Thanks to the development of quantum-dot synthesis^4–7^ and device architectures^8–10^, lab-scale devices with external quantum efficiency from 10 to 20% and emission FWHM ~25 nm have been demonstrated for all the three primary colors for full-color display^10–12^. Moreover, since the technology is highly compatible with printed display, growing number of display manufacturers have included QLED in their roadmaps^13^.

Despite of these achievements, QLED is still in the early stage of research and development. To productize this technology, researchers have to solve the issue of device degradation. Based upon published materials, red, green and blue QLED devices exhibit greatly different operating lifetime, therefore they do not share a universal degradation mechanism. For the ease of comparison, the following referred data are converted to LT50, the time at which the intensity of electroluminescence decays to 50% of its initial value—*L*_0_ = 1000 cd m^−2^, using the raw data and provided acceleration factors. As reported, the lifetime of red QLED has well exceeded 3000 h^11,12,14,15^; green devices are behind but still capable of 1000 h^14^; blue QLED is the worst among the three, delivering lifetime just over 20 h^12,14,16^. Comparing with a rival technology, e.g., printed organic light-emitting diodes (OLED), QLED is far behind because inkjet-printed OLED have reached LT95 (*L*_0_ = 1000 cd m^−2^) of 7000 h for red, 9000 h for green, and 500 h for blue^17,18^.

QLED devices have been using device structures developed for OLED, but their degradation mechanism is worth separated study due to the difference in materials. Unlike organic emitters, the ionization potentials and electron affinities of light-emitting QDs cannot be consistently characterized by existing methods^10,19,20^. Photo-electron spectroscopy, with an analysis depth about 1 nm^21^, has difficulty in resolving the electronic structure of QDs with core-shell or gradient-alloyed nanostructures. Currently, QLED devices are mostly formed with a structure of four functional layers plus a pair of electrodes. The highest occupied molecular orbitals (HOMOs) of hole-transporting layer (HTL) materials, which were designed for OLED, cannot match the deep-lying valence-band maximum (VBM) of Cd-containing QDs. Besides that, the effect of hole mobility and material stability should be considered. For example, poly(9-vinlycarbazole) (PVK, hole mobility *μ*_h_ ~ 10^−6^ cm^2^ V^−1^ s^−1^)^22^ is widely used in blue devices for high quantum efficiency^12,23^, however, due to the very low carrier mobility and the instability of C–N bond^24^, it is known for the negative impact on operating lifetime^23^. In contrast, poly (9,9-dioctylfluorene-co-*N*-(4-(3-methylpropyl))diphenylamine) (TFB) has better stability and hole mobility (*μ*_h_ > 10^−4^ cm^2^ V^−1^ s^−1^). Currently, the best lifetime of blue QLED is achieved using TFB as HTL despite of the mismatch of VBM^14,16^. Electron-transporting layers (ETL) are commonly made of zinc oxide nanoparticles (NPs)^9–11^. Based upon the reported data, ZnO NPs can efficiently inject electrons into the red QDs shelled by CdS^10,11^ or composed of a gradient-alloyed structure (Cd_1−x_Zn_x_Se_1−y_S_y_)^9,12^. For green and blue QDs, which are commonly terminated with ZnS at surface, the electronic property is less reported. Without experimental data, reported band diagrams cannot agree on whether an Ohmic or Schottky contact is formed between ZnO and QDs^25–27^. As the electron affinity decreases in the sequence of red, green and blue QDs^28^, the injection of electron may be a major difference between blue and red devices. So far, reports of QLED mostly cover the topics of luminescence efficiency, lifetime values, and printing methods. To our best knowledge, experimental study on the degradation mechanisms is very limited.

To study the degradation mechanisms, here we choose a popular device structure composed of a transparent anode, a polymeric hole-injection layer (HIL), a polymeric HTL, an emission layer (EML) assembled of red (Cd_1−x_Zn_x_Se_1−y_S_y_) or blue (Cd_1−x_Zn_x_S) dots, an ETL assembled of ZnO NPs, and a top cathode. We choose to focus on blue devices because it is currently the short slab of the full-color QLED display. Red devices are chosen as controls because they outperform the blue ones by more than 100 times. In particular, we choose red (Cd_1−x_Zn_x_Se_1−y_S_y_) and blue (Cd_1−x_Zn_x_S) QDs with a composition gradient from core to surface (x, y increases) which enables highly efficient electroluminescence for all the red, green and blue devices^12^. As blue QDs are chemically similar to those very stable red QDs (Supplementary Fig. 1d), here we assume the short lifetime of blue devices is not primarily due to the chemical instability of QDs.

Red and blue devices will be compared side by side throughout the following section. We begin with the results of lifetime tests. To determine the source of degradation in blue QLED, we then analyze electro-absorption (EA) spectra for the organic HTL, the QD emission layer and the ETL to spot possible space-charge accumulation. Finally we combined the results of EA and capacitance–voltage (*C–V*) characteristics to determine the field distribution across the whole device. Unexpectedly, the slightly degraded HTL, in which oxidized molecules (HTL^+^) are spotted, is barely responsible for device degradation. On the contrary, the ZnO-based ETL is concluded as the major source of operating-voltage rise due to the charge transfer across the QD–ZnO junction and charge accumulation in the ETL. Although the result appears counter-intuitive since oxides are usually more stable than organic materials, the offset of conduction band maximum (CBM) across the ZnO–QD junction causes the unwanted charge transfer.

## Results

### Device performance

**Fig. 1:**
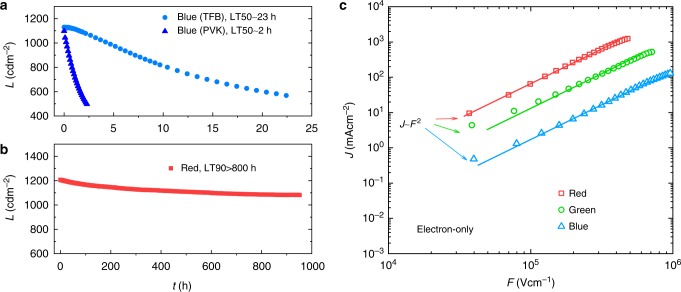
Comparing red and blue devices. **a** Lifetime test results of blue devices. **b** Lifetime results of red devices. **c** *J*–*F* characteristics of electron-only samples. These electron-only devices have a structure of ITO/ZnO/QDs/ZnO/Al

Figure 1 shows the result of lifetime tests. Blue devices with TFB HTL show LT50 (*L*_0_ = 1000 cd m^−2^) of 23 h which make sure the devices do not significantly degrade during our EA and *C–V* measurements. As mentioned, the poor lifetime of PVK device (LT50 ~ 2 h, *L*_0_ = 1000 cd m^−2^) is attributed to the instability of PVK. In comparison, the red device with TFB HTL shows a lifetime (LT90, *L*_0_ = 1000 cd m^−2^) more than 800 h, indicating the primary degradation mechanism is different between red and blue devices. Other regular characterization results of QLED are summarized in Supplementary Fig. 1.

Despite of the same device structure, red and blue QDs have different CBM and VBM values. Figure 1c shows the *J*–*F* characteristics of electron-only devices. Obviously, the sample with red dots has much higher electron current than the sample with blue dots, indicating a large difference of electron-injection barrier. In contrast, the hole injection from TFB to QDs does not follow the same trend (Supplementary Fig. 1c). These results suggest the charge balance in blue devices is poor, but they cannot accurately reflect the situation in a real QLED device. In the following, we decide to apply more powerful techniques to monitor each functional layer of red and blue devices before and after lifetime tests.

### Hole-transporting layer

From the study of OLED, we learned that a hole-injection barrier results in space-charge accumulation and operating-voltage rise in organic HTLs^29,30^. Due to the large ionization potentials, the VBM of Cd-containing QDs cannot be matched by the HOMOs of the HTL materials designed for OLED, therefore the HTL is a possible source of degradation. In this part, we choose to first discuss TFB which currently enables competitive lifetime results (LT50 ~ 23 h, *L*_0_ = 1000 cd m^−2^, Fig. 1a)^16^. The discussion of another commonly used HTL (PVK) is presented in supplementary information.

**Fig. 2:**
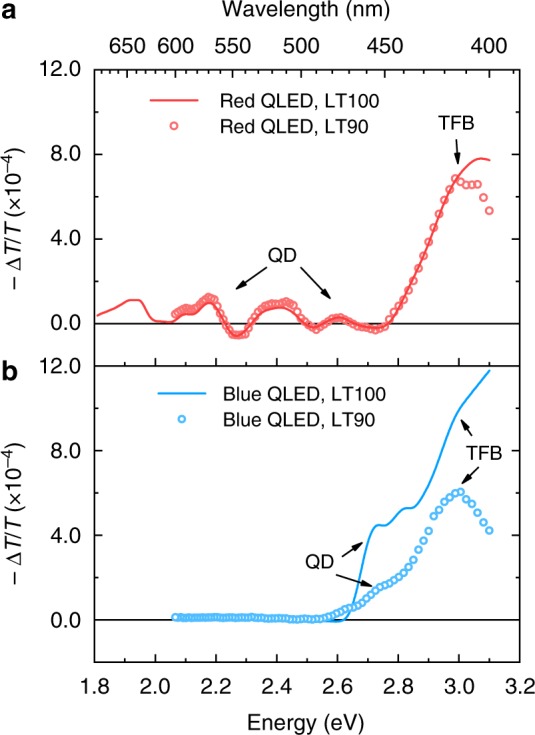
Electro-absorption spectra. **a** In-phase spectra of standard red devices (ITO/PEDOT:PSS/TFB/red quantum dots (QD)/ZnO/Al). **b** In-phase spectra of standard blue devices (ITO/PEDOT:PSS/TFB/blue QD/ZnO/Al). The measurements are carried out using a DC bias of −1 V, AC amplitude of 0.1 V. For each device, the spectrum of electro-absorption is taken before and after the lifetime test. Arrows mark contribution from the hole-transporting layer and QD

**Fig. 3:**
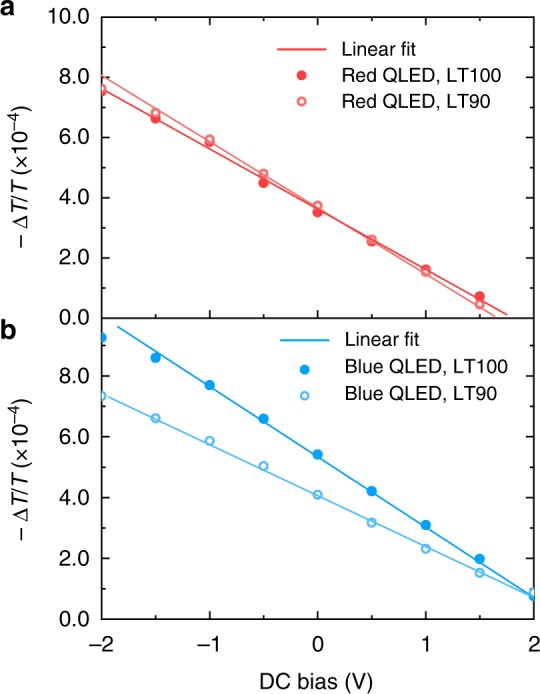
Electric field across the hole-transporting layer. **a** Bias-dependent electro-absorption signals of TFB in standard red devices. **b** Bias-dependent electro-absorption signals of TFB in standard blue devices. The probing wavelength is fixed at 415 nm for TFB; The DC bias determines the internal electric field. Each device is measured before and after the lifetime test

To study HTL, we measured EA spectra for red and blue QLED devices before and after lifetime tests. As seen in Fig. 1b, the red devices made and tested in the same batch exhibit LT90 (*L*_0_ = 1000 cd m^−2^) over 800 h. In the EA test, the sample was placed under reversed bias to avoid charge injection. As shown in Fig. 2a, the electro-absorption of TFB ranges from 400 to 450 nm. The signal at longer wavelengths is from red QDs. Comparing the LT100 and LT90 device, we find them resemble very similar spectra except the TFB region, which will later be attributed to the emergence of TFB^+^. In Fig. 3a, the in-phase signal scales linearly with the applied DC bias, suggesting a typical Stark effect of TFB which is described as

1 $${\mathrm{\Delta }}\alpha (\lambda ) = - \frac{1}{d} \cdot \frac{{{\mathrm{\Delta }}T(\lambda )}}{{T(\lambda )}} \propto {\mathrm{Im}}\chi ^{(3)} \cdot [E(t)]^2 \\ = \, {\mathrm{Im}}\chi ^{(3)} \cdot \left( {2E_{{\mathrm{dc}}}E_{{\mathrm{ac}}}{\mathrm{sin}}\,\omega t + \frac{1}{2}E_{{\mathrm{ac}}}^2{\mathrm{cos}}\,2\omega t} \right)$$

wherein *λ* is the wavelength of the probing light; *α* is the absorption coefficient; *T* is the transmission; Δ*T* is change of transmission due to electric field; *χ*^(3)^ is the imaginary part of the third-order susceptibility of the probed material^31^. A sinusoidal reference field $\left( {E_{{\mathrm{ac}}}{\mathrm{cos}}\,\omega t} \right)$ is superimposed upon the DC bias (*E*_dc_) for modulation and low-noise detection.

As seen in Fig. 1a, the blue devices made and tested in the same batch show LT90 (*L*_0_ = 1000 cd m^−2^) around 4.5 h and LT50 (*L*_0_ = 1000 cd m^−2^) of 23 h. Figures 2b and 3b, respectively, shows the EA spectra and its bias dependence (wavelength fixed at 415 nm) of blue QLED devices. The EA signal from blue QDs ranges from 430 to 470 nm which is the energy of optical bandgap. Similar to what observed in the degraded red QLED device, the in-phase signal from TFB, 415 nm in this case, is relatively strengthened, resulting in a red-shift of this feature. Spectrum change like this has been reported for small molecule OLED wherein a portion of HTL molecules are permanently oxidized (HTL^+^) after lifetime test due to the non-Ohmic contact between HIL and HTL^29^. Since the optical transition energy of HTL^+^ is smaller that of the corresponding HTL molecule, the resultant EA spectrum appears to be red shifted comparing to that of a LT100 sample. In our test, the concentration of space-charge is modulated by the reference bias *V*_ac_ and therefore it contributes charge-modulated signals in addition to the Stark shift^29,32,33^:

2 $${\mathrm{\Delta }}\alpha = - \frac{1}{d} \cdot \frac{{{\mathrm{\Delta }}T}}{T} = n \cdot \sigma$$

wherein *n* is the concentration; *σ* is the absorption cross section of the charged species. Per Eq. 2, the charge-modulated signal is proportional to *n*, thus a heavily degraded HTL layer may exhibit a very different spectrum from that of a fresh sample. Also, since space charges cannot stay in-phase with the modulating bias—*V*_ac_, the growth of quadrature signal and a non-linear voltage dependence should occur simultaneously. Reviewing the data in Fig. 2, we find that although the emergence of TFB^+^ can be spotted, the quadrature signal due to TFB degradation is negligible (see Supplementary Fig. 2 and the arrowed part in Supplementary Fig. 4a). Therefore, the in-phase signal from TFB remains linear as a function of applied bias (Fig. 3a, b), suggesting typical Stark effect with negligible charge-modulation.

In addition to spectra analysis, the signal strength of the Stark effect can be used to study the electric-field distribution across the multilayered devices^34^. As shown in Figs 2a and 3a, the signal strength of TFB’s Stark effect increases slightly after the LT90 test for the red device, suggesting small voltage rise across the HTL. Per Eq. 1, the contribution of *E*_dc_ is in the 1st harmonic portion. When the HTL reaches flat-band condition, the voltage consumption increases gently from ~1.6 to ~1.7 V after the LT90 test, suggesting mild degradation in the HTL. In comparison, as shown in Figs 2b and 3b, the field distribution in the HTL changes differently after the LT90 test. First, TFB’s signal strength decreases significantly after the LT90 test of the blue device, indicating reduced voltage consumption across the HTL. For a degraded blue QLED device, such voltage drop in the HTL has to be over-compensated by the voltage rise across other functional layers, i.e., the QD EML and the ZnO ETL, because the operating-voltage of the whole device increases significantly. Second, as marked by the intercept on the *x*-axis, the voltage bias at which the HTL reaches flat-band condition is almost unchanged despite of the fast degradation, again confirming that the HTL is barely degraded after the LT90 test. The result is also consistent with our finding that the hole injection from TFB to red and blue QDs is not significantly different (Supplementary Fig. 1c).

Combining Figs 2 and 3, the emergence of TFB^+^ in the HTL can explain the slow degradation (LT90 > 800 h) of red QLED devices. Further effort to improve the lifetime of red QLED should focus on the HTL–QD junction^15^. For the blue QLED, the conclusion depends on specific HTL materials. When the HTL is made of PVK, although the Stark shift of PVK (*E*_g_ ~ 3.6 eV) is out of the detection range (Supplementary Fig. 4b and c), the incorporation of PVK reduces the operating lifetime (LT50 ~ 2 h, Fig. 1a). When TFB is used instead, LT50 is improved to 23 h. Nevertheless, our results show that the luminance decay is not primarily determined by the degradation of HTL. As to be shown in the next part, other degradation mechanisms should be considered.

### Electron-transporting layer and emission layer

ZnO has a bandgap energy over 3.4 eV, which is greater than our limit (3.1 eV) for low-noise detection, therefore a direct measurement of ZnO’s Stark shift cannot be done. In addition, the minimum-required photon energy for persistent electron photoconductivity (PPC, or persistent increase of capacitance) of ZnO is reported to be 3.1 eV^35^, therefore charge-modulated signal due to PPC effect is not expected either. We decided circumvent the issue by measuring the EA response of QDs and finishing the analysis using capacitance-voltage characteristics. To conclude the effect of ZnO using EA experiments, we included not only QLED devices with regular structures, but also two sets of specially designed samples—one with a pair of electrodes sandwiching a single layer of (red or blue) QDs and the other with electrodes sandwiching a (red or blue) QD layer plus an additional layer of ZnO.

**Fig. 4:**
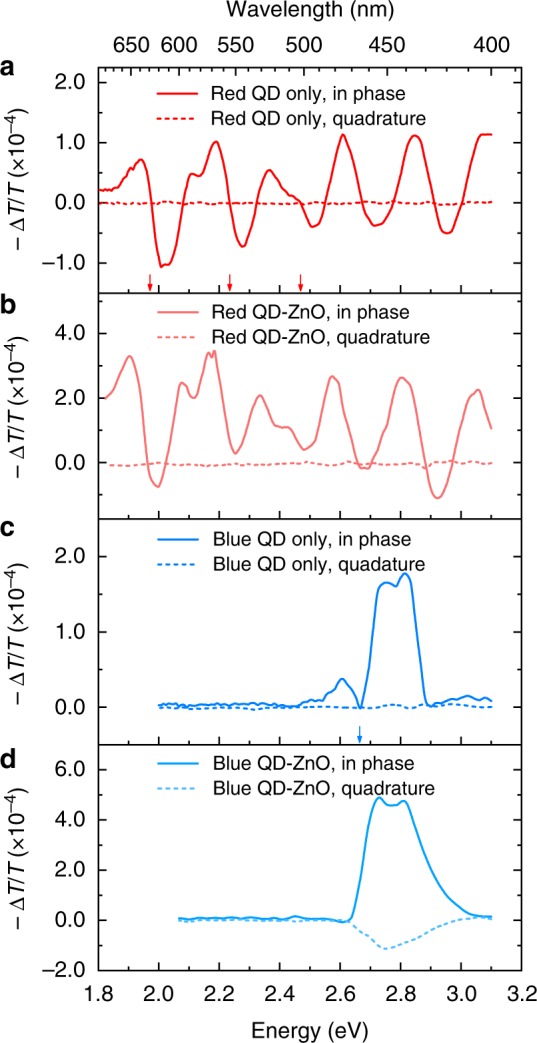
The quantum-dot–ZnO junction. **a** Electro-absorption (EA) spectra (in-phase and quadrature) of the sample with a structure of ITO/red (Cd_1−x_Zn_x_Se_1−y_S_y_) quantum dots (QDs)/Al. **b** EA spectra (in-phase and quadrature) of the sample with a structure of ITO/red (Cd_1−x_Zn_x_Se_1−y_S_y_) QDs/ZnO/Al. **c** EA spectra (in-phase and quadrature) of the sample with a structure of ITO/blue (Cd_1−x_Zn_x_S) QDs/Al. **d** EA spectra (in-phase and quadrature) of the sample with a structure of ITO/blue (Cd_1−x_Zn_x_S) QDs/ZnO/Al. The spectra are obtained using a DC bias of −1 V, AC amplitude of 0.1 V. Arrows mark the wavelength at which in-phase signal reaches zero and they also match excitonic peaks in linear spectra (Supplementary Fig. 1). Relevant discussion is detailed in Supplementary Note 4

The EA spectra of red (Cd_1−x_Zn_x_Se_1−y_S_y_) QDs without and with the addition of a ZnO ETL are plotted in Fig. 4a, b, respectively. Similar to single-compound QDs, the feature at the optical bandgap (1st excitonic level) is due to the Stark shift of QDs^36,37^. As detailed in supporting materials (Supplementary Fig. 5), additional oscillating features at shorter wavelength are attributed to higher excitonic levels^38–40^. As seen, the incorporation of ZnO does not change the profile of in-phase spectra. The difference in the signal strength is due to the increase of built-in potential (*V*_bi_). For both samples, the quadrature signal is negligible throughout the spectra, indicating that the junction between red (Cd_1−x_Zn_x_Se_1−y_S_y_) QDs and ZnO cannot generate charges for electric-field modulation. With the probing wavelength fixed at 570 nm, at which the feature is attributed to Stark shift, the bias-dependent in-phase signal is plotted in Supplementary Fig. 3. The amplitude scales linearly with the applied bias, suggesting negligible charge-modulation effect.

**Fig. 5:**
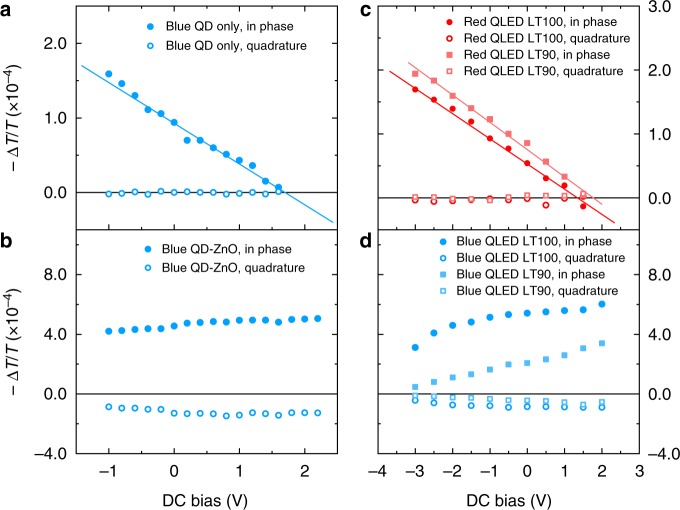
The origin of charge modulation. **a** DC bias-dependent elecctro-absorption (EA) signal of the sample with a structure of ITO/blue (Cd_1−x_Zn_x_S) QDs/Al. **b** DC bias-dependent EA signal of the sample with a structure of ITO/blue (Cd_1−x_Zn_x_S) QDs/ZnO/Al. **c** DC bias-dependent EA signal of a standard red device which is measured before and after the lifetime test. **d** DC bias-dependent EA signal of a standard blue device which is measured before and after the lifetime test. The probing wavelength is 570 nm for red (Cd_1−x_Zn_x_Se_1−y_S_y_) dots and 455 nm for blue (Cd_1−x_Zn_x_S) dots. The straight lines are indicators of the linear relationship between signal strength and applied bias

The EA spectra of blue (Cd_1−x_Zn_x_S) QDs without and with an additional ZnO ETL are plotted in Fig. 4c, d, respectively. Around the optical gap energy of blue (Cd_1−x_Zn_x_S) QDs, two peaks can be resolved in both figures. Comparing to the samples of red QDs, the in-phase signals also increase with the addition of ZnO, however, the EA spectra show very different characteristics. Without the ZnO layer (Fig. 4c), the response of blue QDs is in-phase with the modulating field—showing negligible quadrature signal. With the addition of a ZnO layer (Fig. 4d), the quadrature signal around the optical bandgap grows by more than an order of magnitude due to the charge-modulation effect which is discussed later. The bias dependence measurement at given probing wavelength was further carried out to confirm the result of the spectra analysis. Without ZnO (Fig. 5a), the linear relationship between the in-phase signal and applied bias is maintained with a probe beam at the wavelength of 455 nm, indicating that the EA response is dominated by the effect of Stark shift. With the addition of ZnO (Fig. 5b), the −Δ*T*/*T* vs. bias relationship is completely irrelevant to Eq. 1. Instead, both in-phase and quadrature signals (at 455 nm) resemble characteristics of a leaky capacitor wherein the amount of charges for field modulation reduces when *E* field increases. Therefore, the addition of a ZnO layer to blue QDs introduces strong charge modulation effect.

The contrasting effect of ZnO ETL on red and blue QDs is also observed in standard QLED devices. First, as seen in the spectra of Supplementary Fig. 2, red devices show no sign of quadrature signals before or after the lifetime tests, while blue devices with either TFB or PVK HTLs (Supplementary Fig. 4a and c) shows strong quadrature spectra around the wavelength of 460 nm, which is consistent with Fig. 4d. Second, as shown in Fig. 5c, d, the bias dependence of QDs’ EA signal is measured for LT100 and degraded QLED devices. For the red QLED devices after the LT90 test (Fig. 5c), the signal strength of red QDs increases slightly comparing to that of the LT100 control device. Similar to the observation from Fig. 3a, the voltage across the red QD layer does not increase significantly during degradation, which is consistent with the result that red devices experience marginal operating-voltage rise after the LT90 test. The linear −Δ*T*/*T* vs. bias relationship and negligible quadrature signal confirms there is no charge modulation across the QD–ZnO junction in red QLED devices. In Fig. 5d, the bias dependence of in-phase and quadrature signal is consistent with that in Fig. 5b, demonstrating strong charge modulation in the standard blue QLED device at both negative and positive bias. The corresponding spectra taken at *V*_dc_ = −1 V are shown in Supplementary Fig. 4a. Here, we need to point out that, although the charge modulated signal is linked with device degradation, the transfer function from |−Δ*T*/*T*_quadrature_| to the luminance degradation cannot be established at this stage because the amount of charges available for field modulation is cumulatively determined by the internal electric field across the QD–ZnO junction and the generation of charged species. In heavily degraded samples, the internal field at given bias increases significantly due to the increased operating-voltage. As a result, the −Δ*T*/*T* vs. bias curve is translated along the voltage axis by +2 to +3 V (Fig. 5d), which is consistent with the operating-voltage rise during the lifetime test, leading to reduction of quadrature signal at given bias.

Summarizing the results of Figs 4 and 5 into Supplementary Table 1, we notice that the charge modulation features, either in the spectra (Fig. 4d) or bias dependence plot (Fig. 5b, d), only occur to the samples with both blue QDs and ZnO ETL. To explain the origin of charge modulation, electron-transfer from blue (Cd_1−x_Zn_x_S) QDs to ZnO NPs should be discussed. The charge-transfer process between QDs and oxides is known as a charge-generation mechanism in QD photovoltaic devices^41^. For QLED, the QD–oxide junction is usually linked with single-dot emission intermittency (known as blinking), Auger recombination and reduction of photoluminescence^42,43^. In specific, n-type materials like ITO^44^, TiO_x_ nanoparticles^45,46^ and p-type materials like NiO_x_ nanoparticles^47^ can suppress the blinking of single dot at the expense of the photoluminescence quantum yield. Proposed blinking dynamics begin with electron (or hole) transfer from QD to n-type (or p-type) oxide (nanoparticles) and leave the QD in the positively (or negatively) charged off state; a back-transfer of charge is needed to turn the single dot on. For the case of QD–ZnO, reduction of photoluminescence was also observed and the model of electron transfer was proposed correspondingly^10,11^. As known, although the accurate value remains arguable, the electron affinity of blue (Cd_1−x_Zn_x_S) QDs is significantly smaller than that of red QDs due to the increased bandgap energy and the small value of $m_{\mathrm{e}}^ \ast /m_{\mathrm{h}}^ \ast$, wherein $m^ \ast$ represents effective mass. The resultant CBM offset across the QD–ZnO junction is reflected by the *J–F* characteristics of electron-only devices in Fig. 1c. From these curves, although we cannot extract the barrier-height between QD and ZnO using models like Richardson–Schottkly transport^48^ and Arkhipov’s theory^49,50^ which consider one metal–semiconductor junction, band diagram in Supplementary Fig. 6 suggests that ZnS at surface leads to substantial CBM offset which is enough to result in electrons transfer from blue dots to ZnO. For the same reason, the back-transfer of electrons is not favored, eventually resulting in charge accumulation in ZnO. Except for the offset of CBM energy, such charge transfer and trapping can be further facilitated by the structure and size of QDs. For example, the QDs with gradient-alloyed structure have a soft confinement of electrons which increases the chance of electron tunneling to the surface of QDs. Also, the blue QDs has a similar diameter to ZnO NPs (~5 nm), therefore ZnO NPs can get closer proximity to blue QDs and increase the chance of charge transfer. Therefore, we can describe the charge modulation effect as following. First, upon excitation of a blue dot, subsequent charge transfer leaves blue QDs positively charged and ZnO negatively charged, and their carrier concentration is modulated by the reference bias (*V*_ac_). Then, the accepted electrons in ZnO experience trapping and de-trapping, which slows down the response of charges to the modulating bias and gives rise to the strong quadrature signals.

Through the spectra analysis (Fig. 4c, d) and the study of bias dependence (Fig. 5), we conclude that the insertion of a ZnO NPs layer results in charge transfer across the blue QD–ZnO interface as long as the blue QD is excited, resulting in strong charge-modulated signals. In the following, it shows that the charge transfer process is responsible for the voltage rise in ETL. Again, these effects are only observed in samples with both blue QDs and ZnO. The consistence between EA and *C–V* results should explain the different degradation mechanisms between red and blue QLED devices.

### Charge balance

*C–V* is an effective way to evaluate the charge balance in QLED. To minimize the contribution from (deep level) traps, a modulating frequency at 100 kHz is used. We are able to obtain smooth curves even from heavily degraded devices. Under reversed bias, the whole device can be roughly treated as three planar capacitors connected in series, with each capacitor corresponding to the depleted HTL, QD layer and ETL^51^. When the applied bias turns to positive, a charge-unbalanced device will let the fast carrier inject first, i.e., electrons (holes) in red (blue) QLED, resulting in capacitance rise. The applied voltage concentrates on the transporting layer of the slow carrier, i.e., HTL (ETL) in red (blue) QLED, until the bias is high enough to cause efficient radiative recombination and capacitance drop. Given that both red and blue QLED are charge-unbalanced systems, measuring *C–V* is an adequate method to evaluate the field distribution.

**Fig. 6:**
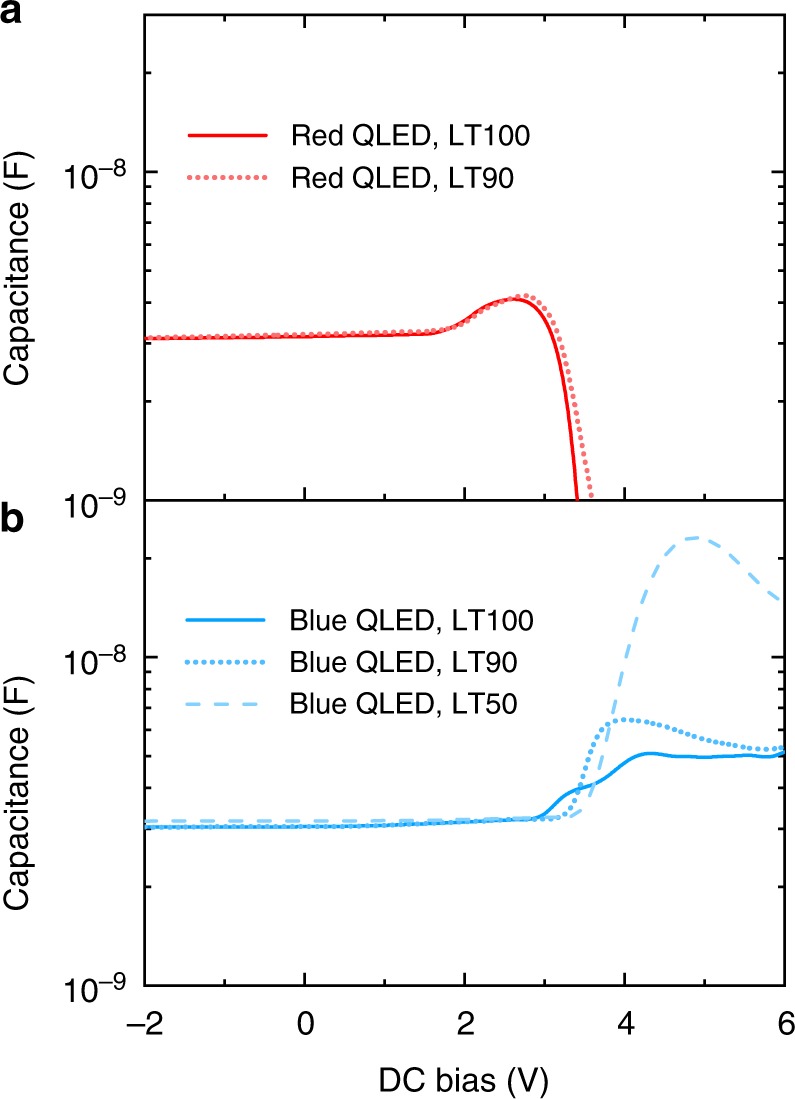
Internal field distribution and charge balance. **a** The capacitance-voltage (*C–V*) characteristics of a standard red device. **b** The *C–V* characteristics of a standard blue device. In the measurements, the modulating frequency is 100 kHz, and the modulating amplitude is 20 mV. Each device is measured before and after the lifetime test

The *C–V* results of standard red and blue QLED devices are plotted in the same semi-logarithmic scale. In Fig. 6a, the capacitance values of red QLEDs stay almost constant until the bias of 1.5 V and then decreases significantly after 2.5 V. The relative constant value of 3.2 nF roughly matches the geometric capacitance of HTL (4.2 nF), QD layer (>20 nF), and ETL (ZnO, 25 nF) connected in series. In a red QLED device, electron injection is more efficient than that of holes, therefore the capacitance rise at 1.8 V means that the HTL (TFB) is the only layer remaining depleted and other layers have reached flat band and charge injection. In fact, the peak capacitance value of 4.0 nF agrees with the geometric capacitance of a 40 nm-thick TFB layer. The bias at which capacitance starts to decrease sharply marks the occurrence of efficient radiative recombination. A fresh red device has a luminance turn-on voltage of at 1.6 V, however, reasonable electroluminescence efficiency (~15 cd A^−1^) is not reached until a bias of 2.4 V^12^, which is consistent with our *C–V* data. After the LT90 test (Fig. 5c), the red device experiences a slight increase of capacitance-transition voltage for about 0.1 V due to the same voltage rise in the HTL. Combining the result with that in Fig. 3a, we can conclude that slow degradation of red QLED originates from the mild degradation of HTL.

The *C–V* results of blue QLED devices (TFB as HTL) are shown in Fig. 6b. Under reversed bias, the capacitance is almost the same as that of the red device due to similar device structure and materials. For the LT100 device, capacitance starts to increase at 2.8 V, and the peak value reaches 5.2 nF. At a modulating frequency of 100 kHz, such an increase of capacitance is unlikely due to a depleted organic HTL. In fact, assuming the injection of holes is more efficient than that of electrons, the HTL in the blue device reaches the flat-band condition in prior to the ETL, leaving voltage concentrated across the ETL. This is consistent with the result of single-carrier devices (Fig. 1c and Supplementary Fig. 1c) and the results of EA measurements (Fig. 2). When the blue device degrades, the *C–V* characteristics change significantly. In Fig. 6b, the capacitance of the LT90 sample turns on at 3.2 V and peaks at 6.6 nF. For the LT50 sample, the turn-on voltage and peak capacitance further increases to 3.3 V and 24 nF, respectively. The big shift of transition voltage is an evidence of significant charge accumulation due to inefficient charge transport, which is similar to the case of small-molecular OLED^51,52^. Further, the very high capacitance confirms that the injection holes is more efficient that electrons, and the charge accumulation occurs in the ETL. Here, with a bulk dielectric constant (100 kHz) of *ε*_r_ ~ 30^53^, volume fraction of solids ~0.7, and a device area of 4 mm^2^, the geometric capacitance of a 30 nm-thick ZnO NPs layer is roughly 25 nF, which is in good agreement with the peak capacitance of our LT50 sample. When the sample is biased to its peak capacitance, the ZnO ETL is the only functional layer that remains depleted. In blue QLED devices, the large barrier for electron injection and the charge transfer at the QD–ZnO interface result in significant charge accumulation during continuous driving, leading to increased voltage drop across the ETL and device degradation. For blue devices with PVK HTL (Supplementary Fig. 7), the carrier injection of holes and electrons are both inefficient, resulting in even field distribution. Since the degradation due to PVK is much faster than that due to ETL, the increase of capacitance is not very significant before the failure of device.

## Discussion

To pinpoint the mechanism of device degradation in red and blue QLED, measurements of electro-absorption and capacitance–voltage characteristics are carried out. First, through the analysis of EA spectra, the emergence of HTL^+^ was spotted in both red and blue devices after their lifetime tests, but the operating-voltage rise across the HTLs is insignificant. Therefore, HTL is responsible the slow degradation of red devices, however, it is not the culprit for the poor lifetime of blue devices. Next, evident by the results of electro-absorption and capacitance measurements, the voltage drop across the ETL (ZnO) was found to increase significantly after the degradation of blue QLED devices. Upon the formation of QD–ZnO junction, ZnO NPs become the acceptor for the excited electrons in blue (Cd_1−x_Zn_x_S) QDs under the driving force of CBM offset, resulting in charge transfer and accumulation. Finally, capacitance–voltage characteristics of blue QLED confirm that the voltage consumption across the ETL increases significantly during the lifetime test. To solve the lifetime issue, it is necessary to develop ETL and QD materials that enable smaller CBM offset.

## Methods

### Preparation of materials

The blue (Cd_1−x_Zn_x_S), and red (Cd_1−x_Zn_x_Se_1−y_S_y_) quantum dots used here were prepared according to methods reported previously in the literature, with appropriate modifications^54,55^. ZnO nanoparticles were synthesized using a solution-precipitation process as reported in the literature^9^. For a typical synthesis, a solution of zinc acetate in dimethyl sulphoxide (DMSO, 0.5 M) and 30 ml of a solution of tetramethylammonium hydroxide (TMAH) in ethanol (0.55 M) were mixed and stirred for 1 h in ambient air, then washed and dispersed in ethanol for device fabrication.

### Device fabrication and characterization

Standard QLED devices were fabricated by spin coating on glass substrates that were commercially pre-coated with an indium-tin oxide anode (sheet resistance ∼25 Ω □^−1^). The substrates were cleaned consecutively in ultrasonic baths of deionized water, acetone and 2-propanol for 15 min each, and were then exposed to an ultraviolet ozone ambient for 15 min. The substrates were spin-coated with PEDOT:PSS (AI 4083) and baked at 150 °C for 15 min in air. The coated substrates were then transferred into a nitrogen-filled glove box for spin coating of layer of TFB, quantum dots and ZnO nanoparticles. TFB was purchased from American Dye Source, and used as supplied. The TFB layers were spin-coated at 4000 r.p.m. for 30 s using an 8 mg ml^−1^ solution in chlorobenzene, followed by baking at 150 °C for 30 min. Quantum dots and ZnO nanoparticle layers were then spin-coated and baked at 70 °C for 30 min. The optimized quantum-dot layer thicknesses were ∼16 nm for red (15 mg ml^−1^, 2500 r.p.m.), ∼20 nm for green (18 mg ml^−1^ quantum-dot solution, 2000 r.p.m. spin speed), and ∼20 nm for blue (18 mg ml^−1^, 2000 r.p.m.), as determined from an efficiency comparison of devices with various quantum-dot layer thicknesses. Depending on the emitting wavelength, the thickness of the ZnO is controlled between 30 and 65 nm by changing the solution concentration and spin speed. Finally, the multilayer samples were loaded into a high-vacuum chamber (base pressure ∼1 × 10^−7^ Torr) for deposition of an Al cathode (100 nm), patterned by a shadow mask to form devices with an active area of 4 mm^2^. All devices were encapsulated in commercially available ultraviolet-curing epoxy and cover glass. For the lifetime test, the encapsulated samples were measured under ambient conditions using a commercialized lifetime test system (Guangzhou New Vision Opto-electronic Technology Co. Ltd.).

### Electro-absorption

The samples were kept inside a cryostat (Janis VPF-100, liquid nitrogen) with a pressure of 10^−3^ Torr. A monochromatic parallel beam probes the sample through the ITO side with an incident angle of 45  and is reflected by the back electrode. To modulate the internal electric field for low-noise detection, a sinusoidal voltage with frequency of 1 K Hz was superimposed to the DC bias, producing a periodical bias in the form of $V(t) = V_{{\mathrm{dc}}} + V_{{\mathrm{ac}}}{\mathrm{sin}}\,\omega t$, wherein *ω* is the modulating frequency. When measuring the electro-absorption spectra, the DC bias is negative to avoid carrier injection into the devices. The back-reflected signal got further absorbed by the organic layer before being collected by the photodetectors. Calibrated silicon and germanium photodetectors were used to detect the reflected signal. A current amplifier and a lock-in amplifier (SR830) were connected to the detector and locked to 1st harmonic frequency for low-noise measurement. The final signal was the ratio of the signals with and without the modulation of *V*_ac_. To lower the noise, the time constant of the lock-in was set at 1 s. Each data point is averaged from 16 measurements.

### Capacitance–voltage characterization

Measurements were carried out with an Agilent 4282A precision LCR meter and the data was automatically acquired by a computer. To avoid the effect of trapped charges, the modulating frequency and the modulating amplitude is 100 kHz and 20 mV, respectively.

## Supplementary information

Supplementary Information

## Data Availability

Source data are available from corresponding authors upon request.

## References

[1] Shirasaki Y, Supran GJ, Bawendi MG, Bulović V (2013). Emergence of colloidal quantum-dot light-emitting technologies. Nat. Photon..

[2] Supran GJ (2013). QLEDs for displays and solid-state lighting. Mrs. Bull..

[3] Dai X, Deng Y, Peng X, Jin Y (2017). Quantum-dot light-emitting diodes for large-area displays: towards the dawn of commercialization. Adv. Mater..

[4] Murray CB, Norris DJ, Bawendi MG (1993). Synthesis and characterization of nearly monodisperse CdE (E = sulfur, selenium, tellurium) semiconductor nanocrystallites. J. Am. Chem. Soc..

[5] Peng X (2000). Shape control of CdSe nanocrystals. Nature.

[6] Peng ZA, Peng X (2001). Formation of high-quality CdTe, CdSe, and CdS nanocrystals using CdO as precursor. J. Am. Chem. Soc..

[7] Chen O (2013). Compact high-quality CdSe–CdS core–shell nanocrystals with narrow emission linewidths and suppressed blinking. Nat. Mater..

[8] Caruge JM, Halpert JE, Wood V, Bulović V, Bawendi MG (2008). Colloidal quantum-dot light-emitting diodes with metal-oxide charge transport layers. Nat. Photon..

[9] Qian L, Zheng Y, Xue J, Holloway PH (2011). Stable and efficient quantum-dot light-emitting diodes based on solution-processed multilayer structures. Nat. Photon..

[10] Mashford BS (2013). High-efficiency quantum-dot light-emitting devices with enhanced charge injection. Nat. Photon..

[11] Dai X (2014). Solution-processed, high-performance light-emitting diodes based on quantum dots. Nature.

[12] Yang Y (2015). High-efficiency light-emitting devices based on quantum dots with tailored nanostructures. Nat. Photon..

[13] Kovalenko MV, Protesescu L, Bodnarchuk MI (2017). Properties and potential optoelectronic applications of lead halide perovskite nanocrystals. Science.

[14] Qian L (2017). 6-2: *Invited Paper*: Key challenges towards the commercialization of quantum-dot light-emitting diodes. SID Symp. Dig. Tech. Pap..

[15] Cao W (2018). Highly stable QLEDs with improved hole injection via quantum dot structure tailoring. Nat. Commun..

[16] Shen H (2017). Efficient and long-lifetime full-color light-emitting diodes using high luminescence quantum yield thick-shell quantum dots. Nanoscale.

[17] Levermore P (2016). 38‐1: *Invited Paper*: Ink‐jet‐printed OLEDs for display applications. SID Symp. Dig. Tech. Pap..

[18] Yamada T (2017). 57‐2: *Invited Paper*: Latest development of high‐performance OLED material suitable for printing. SID Symp. Dig. Tech. Pap..

[19] Cho KS (2009). High-performance crosslinked colloidal quantum-dot light-emitting diodes. Nat. Photon..

[20] Wood V (2009). Selection of metal oxide charge transport layers for colloidal quantum dot LEDs. ACS Nano.

[21] Hüfner, S. *Photoelectron Spectroscopy: Principles and Applications* (Springer Science & Business Media, 2013).

[22] Lee DH, Liu YP, Lee KH, Chae H, Cho SM (2010). Effect of hole transporting materials in phosphorescent white polymer light-emitting diodes. Org. Electron..

[23] Wang O (2018). High-efficiency, deep blue ZnCdS/Cd_x_Zn_1−x_S/ZnS quantum-dot-light-emitting devices with an EQE exceeding 18%. Nanoscale.

[24] Kondakov DY, Lenhart WC, Nichols WF (2007). Operational degradation of organic light-emitting diodes: mechanism and identification of chemical products. J. Appl. Phys..

[25] Lee KH (2013). Highly efficient, color-pure, color-stable blue quantum dot light-emitting devices. ACS nano.

[26] Kwak J (2012). Bright and efficient full-color colloidal quantum dot light-emitting diodes using an inverted device structure. Nano. Lett..

[27] Shen H (2015). High-efficiency, low turn-on voltage blue-violet quantum-dot-based light-emitting diodes. Nano. Lett..

[28] Anikeeva PO, Halpert JE, Bawendi MG, Bulović V (2009). Quantum dot light-emitting devices with electroluminescence tunable over the entire visible spectrum. Nano. Lett..

[29] Lane PA, Chen S, So F (2011). Electromodulated doping of the hole transport layer in a small molecule organic light-emitting diode. J. Photon Energy.

[30] So F, Kondakov D (2010). Degradation mechanisms in small-molecule and polymer organic light-emitting diodes. Adv. Mater..

[31] Sebastian L, Weiser G (1981). One-dimensional wide energy bands in a polydiacetylene revealed by electroreflectance. Phys. Rev. Lett..

[32] Tsang SW, Chen S, So F (2013). Energy level alignment and sub‐bandgap charge generation in polymer: fullerene bulk heterojunction solar cells. Adv. Mater..

[33] Chen S, Tsang SW, Lai TH, Reynolds JR, So F (2014). Dielectric effect on the photovoltage loss in organic photovoltaic cells. Adv. Mater..

[34] Campbell IH (1997). Measuring internal electric fields in organic light-emitting diodes using electroabsorption spectroscopy. Polym. Adv. Technol..

[35] Lany S, Zunger A (2005). Anion vacancies as a source of persistent photoconductivity in II-VI and chalcopyrite semiconductors. Phys. Rev. B.

[36] Liu X, Iimori T, Ohshima R, Nakabayashi T, Ohta N (2011). Electroabsorption spectra of PbSe nanocrystal quantum dots. Appl. Phys. Lett..

[37] Awasthi K, Iimori T, Ohta N (2014). Integral method analysis of electroabsorption spectra and its application to quantum dots of PbSe. J. Phys. Chem. C..

[38] Wehrenberg BL, Guyot-Sionnest P (2003). Electron and hole injection in PbSe quantum dot films. J. Am. Chem. Soc..

[39] Sargent EH (2005). Infrared quantum dots. Adv. Mater..

[40] Colvin VL, Cunningham KL, Alivisatos AP (1994). Electric field modulation studies of optical absorption in CdSe nanocrystals: dipolar character of the excited state. J. Chem. Phys..

[41] Kamat PV (2008). Quantum dot solar cells. Semiconductor nanocrystals as light harvesters. J. Phys. Chem. C..

[42] Galland C (2011). Two types of luminescence blinking revealed by spectroelectrochemistry of single quantum dots. Nature.

[43] Efros AL, Nesbitt DJ (2016). Origin and control of blinking in quantum dots. Nat. Nanotechnol..

[44] Jin S, Song N, Lian T (2010). Suppressed blinking dynamics of single QDs on ITO. ACS nano.

[45] Jin S, Lian T (2009). Electron transfer dynamics from single CdSe/ZnS quantum dots to TiO_2_ nanoparticles. Nano. Lett..

[46] Hamada M, Nakanishi S, Itoh T, Ishikawa M, Biju V (2010). Blinking suppression in CdSe/ZnS single quantum dots by TiO_2_ nanoparticles. ACS nano.

[47] Wu X, Yeow EK (2010). Charge-transfer processes in single CdSe/ZnS quantum dots with p-type NiO nanoparticles. Chem. Commun..

[48] Sze, S. M. *Semiconductor Devices: Physics and Technology* (John Wiley & Sons, Hoboken, 2008).

[49] Arkhipov V, Emelianova E, Tak Y, Bässler H (1998). Charge injection into light-emitting diodes: Theory and experiment. J. Appl. Phys..

[50] Wolf U, Arkhipov VI, Bässler H (1999). Current injection from a metal to a disordered hopping system. I. Monte Carlo simulation. Phys. Rev. B.

[51] Kondakov DY, Sandifer JR, Tang CW, Young RH (2003). Nonradiative recombination centers and electrical aging of organic light-emitting diodes: direct connection between accumulation of trapped charge and luminance loss. J. Appl. Phys..

[52] Chen S, Jiang X, So F (2013). Hole injection polymer effect on degradation of organic light-emitting diodes. Org. Electron..

[53] Langton N, Matthews D (1958). The dielectric constant of zinc oxide over a range of frequencies. Br. J. Appl. Phys..

[54] Bae WK, Char K, Hur H, Lee S (2008). Single-step synthesis of quantum dots with chemical composition gradients. Chem. Mater..

[55] Bae WK, Nam MK, Char K, Lee S (2008). Gram-scale one-pot synthesis of highly luminescent blue emitting Cd_1− x_Zn_x_S/ZnS nanocrystals. Chem. Mater..

