# Supplementary material for: On the degradation mechanisms of quantum-dot light-emitting diodes
Source: Nat Commun. 2019 Feb 15;10:765. doi: 10.1038/s41467-019-08749-2 (PMC6377672; doi:10.1038/s41467-019-08749-2)
Supplement: Supplementary file 1 — Supplementary Information [file 41467_2019_8749_MOESM1_ESM.pdf]

On the Degradation Mechanisms of Quantum Dot Light-Emitting Diodes

Chen et al.

Supplementary Note 1. QLED, single-carrier devices and QDs

As seen in Supplementary Figure 1a and b, blue devices with TFB and PVK as HTLs show contrasting characteristics in the plots of *L-J-V* and quantum efficiency. In blue devices with PVK as HTL, both hole and electron injection are inefficient. In blue devices with TFB as HTL, hole injection is more efficient than electrons (see Figure 6b, Figure 1c and Supplementary Figure 1c). The poor lifetime of PVK-containing devices is partially related to the inefficient carrier injection. In Supplementary Figure 1c, the hole injection is compared. Since the hole injection from TFB to red and blue QDs are fairly similar. To be noted that, the current in single-carrier devices is not only determined by carrier-injection at the junction we are interested in, but also affected by the resistivity of the blocking layer which is inorganic in electron-only devices but organic in hole-only devices. Therefore, a direct comparison of hole and electron current in corresponding single-carrier devices is misleading. In our views, the evaluation of charge-balance should be conducted on a bipolar device, using techniques like *C-V* to directly probe the electric field distribution across the diode.

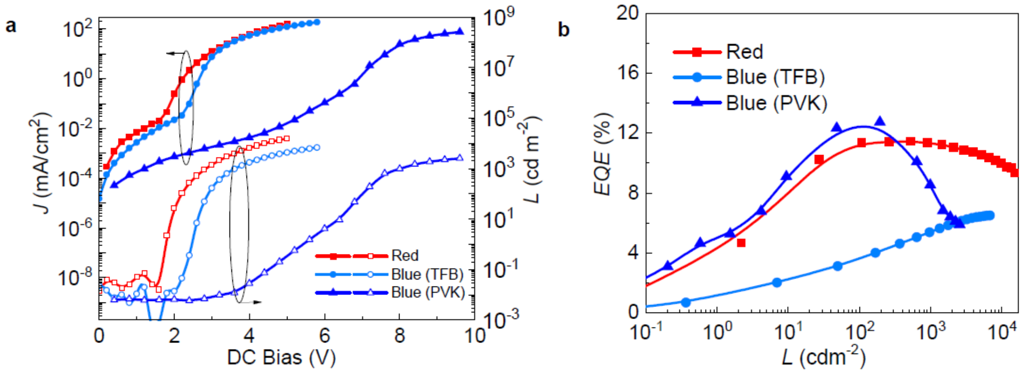

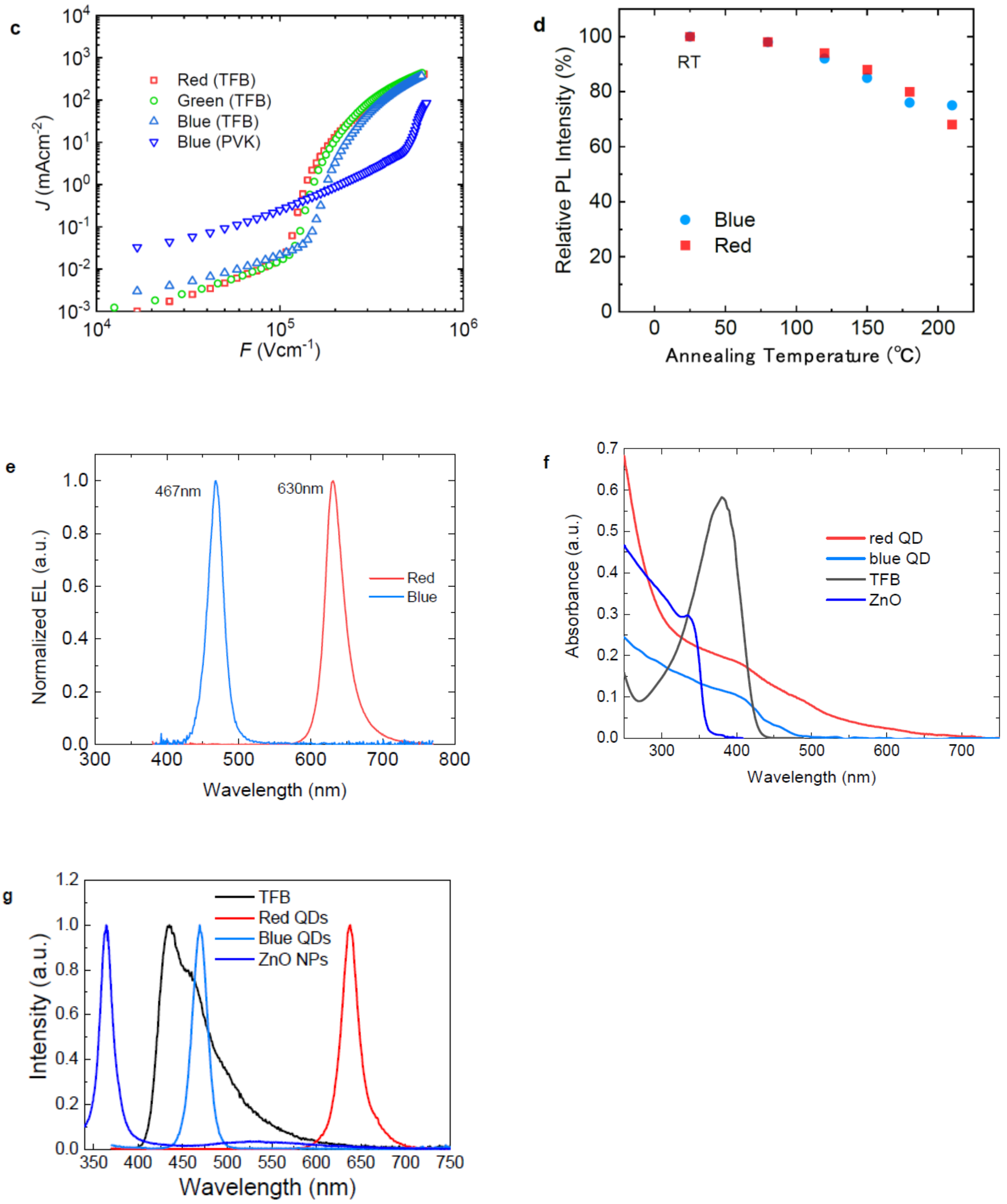

Supplementary Figure 1. Quantum dots and devices. a *L*-*J*-*V* characteristics of quantum-dot light-emitting diodes (QLED) used for lifetime study. **b** External quantum efficiencies of the red and blue QLED devices used for lifetime study; **c** *J*-*F* characteristics of hole-only devices which have a structure of ITO/PEDOT:PSS/HTL/QD/TAPC:HAT-CN/Au; **d** Thermal stability of red and blue QDs. PL intensity is measured from solid thin films. The annealing time is 15 minutes. **e** Normalized EL spectra for red and blue QLEDs. **f** Absorption spectra. **g.** PL spectra of all the materials (measured as thin films) used to compose QLED devices.

Supplementary Note 2. Electro-absorption data of standard red QLED

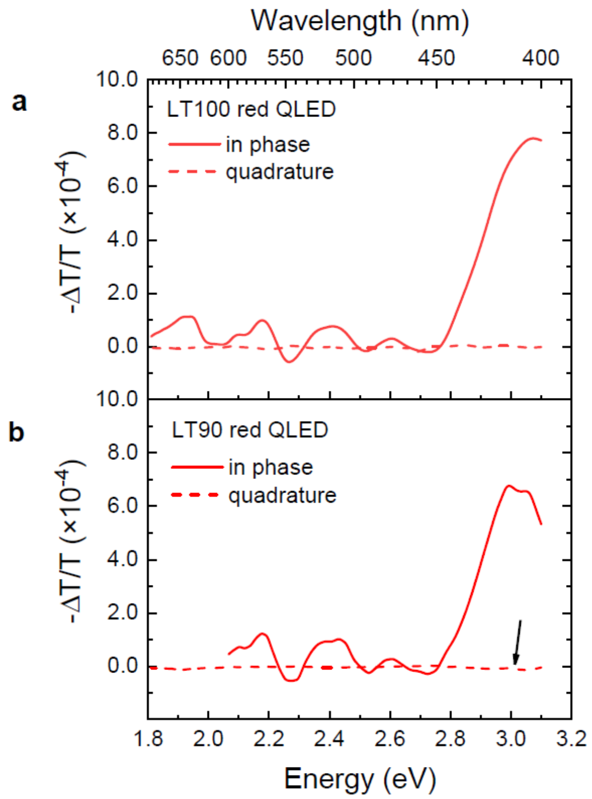

Supplementary Figure 2. **Electro-absorption spectra**. **a** In-phase and quadrature signal of a pristine red device; **b** In-phase and quadrature signal of a red device before and after the lifetime test. The measurements were done using a V_{dc} of -1V, V_{ac} of 0.1 V. The two displayed devices have a device structure of ITO/ PEDOT:PSS/ TFB/ quantum dot/ ZnO/ Al.

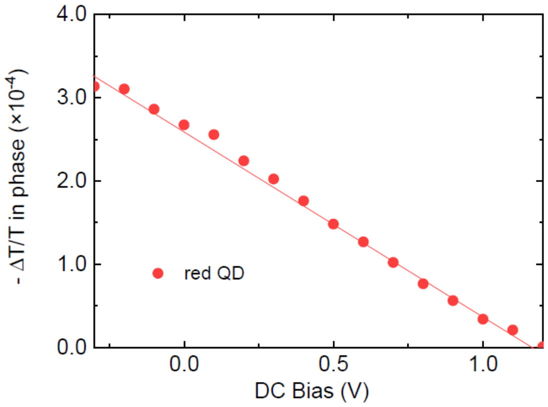

Supplementary Figure 3. **Bias dependent electro-absorption of red quantum dots**. The sample has a structure of ITO/ red ($Cd_{1-x}Zn_xSe_{1-y}S_y$) quantum dots/ ZnO/ Al. The data suggests typical Stark effect and insignificant charge-modulated signals. The probing wavelength is 570 nm.

Supplementary Note 3. Electro-absorption data of blue QLED (TFB or PVK HTL)

After the lifetime test, the charge modulated (quadrature, ~460 nm) signals due to the charge transfer from QDs decreases. As explained in the main text, the absolute value of quadrature signal is not good measure of modulated charges. Although the signal strength of charge modulation follows Equation 2 in main text, the amount of charges available for field modulation is also determined by the internal electric field across the QD-ZnO junction. In heavily degraded samples, the internal field increases significantly at fixed bias due to the increased operating-voltage. As a result, less charges can be modulated. The arrows mark the wavelength at which quadrature signals from TFB+ were expected, if there's any. The samples of Supplementary Figure 4(b) and (c) have a structure of ITO/ PEDOT:PSS/ PVK/ QD/ ZnO/ Al. (b) shows the in-phase spectra and (c) shows the quadrature spectra. Since the bandgap energy of PVK (3.6 eV) is much higher than the cutoff energy (3.1 eV) of the measurement system, PVK does not produce any additional feature. As a result, the spectra are very similar to Figure 4d wherein no HTL materials is included. After the fast degradation of PVK, the in-phase signal decreases due to the voltage-rise in PVK, while the corresponding quadrature signal is almost unchanged because the PVK layer, instead of the ETL, is the primary failure mode in this specific structure. Blue QLED devices with PVK as HTL show high external quantum efficiency (over 15%) but very poor operating lifetime (LT 50 ~2 hours). The underpinning mechanism is that PVK slows down the hole transport and therefore improves the charge balance in the QD emission layer,

resulting in luminance efficiency. However, PVK degrades fast under electrical stress.

Measurements were done using a V_{dc} of -1V, V_{ac} of 0.1 V.

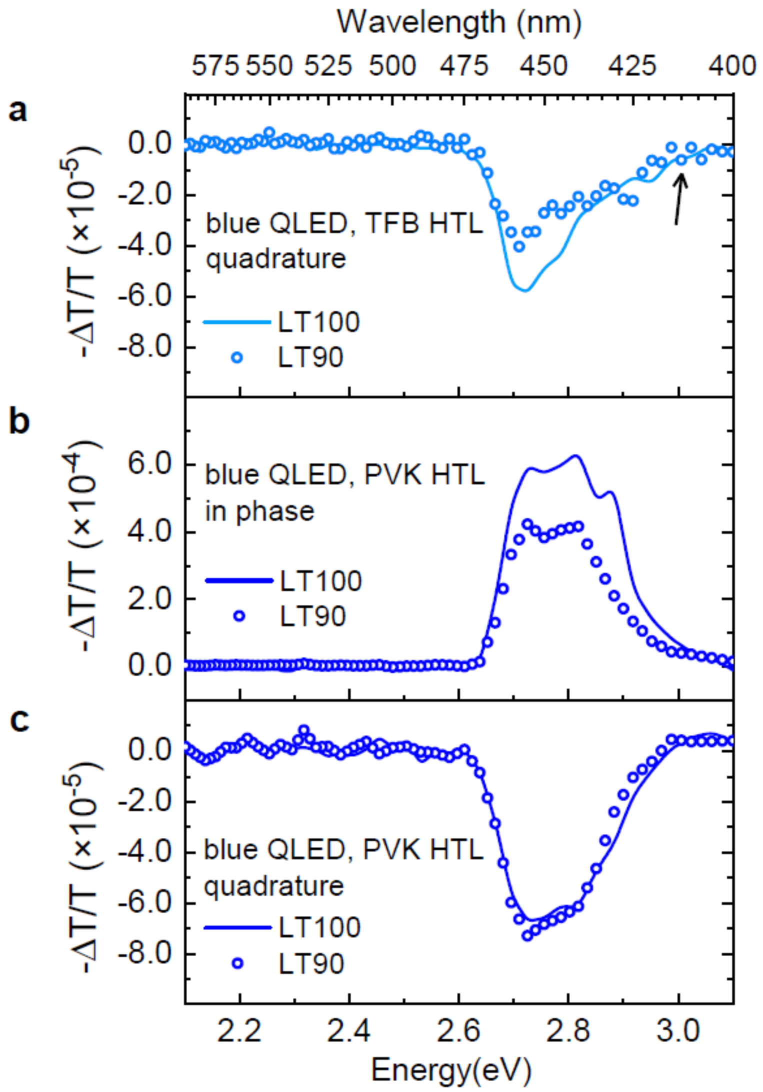

Supplementary Figure 4. **The charge modulation effect**. **a** Quadrature signal of blue devices before and after the lifetime tests. The sample has a structure of ITO/ PEDOT:PSS/ TFB/ quantum dot (QD)/ ZnO/ Al. **b** In-phase signal of blue devices before and after the lifetime tests. The sample has a structure of ITO/ PEDOT:PSS/ PVK/ QD/ ZnO/ Al. **c** Quadrature signal of blue devices before and after the lifetime tests. The sample has a structure of ITO/ PEDOT:PSS/ PVK/ QD/ ZnO/ Al.

Supplementary Note 4. Interpretation of electro-absorption spectra (Figure 4 in main text)

Similar to other excitonic systems, the EA spectra of QDs are due to the Stark shift of excitonic levels, which enables a fitting using the derivatives of linear absorption spectrum. The portion fitted by 1st derivative is due to the change of polarizability ($\Delta\alpha$) between the ground state and excited state. The portion fitted by 2nd derivative is due to the change of electric dipole moment ($\Delta\mu$). Therefore, the EA signal can be described using the following equation:[1,2]

$$\Delta A(\omega) = (fF)^2 \left[A_\chi A(\omega) + B_\chi \omega \frac{\mathrm{d}}{\mathrm{d}\omega}\left(\frac{A(\omega)}{\omega}\right) + B_\chi \omega \frac{\mathrm{d}^2}{\mathrm{d}\omega^2}\left(\frac{A(\omega)}{\omega}\right)\right] \quad (1)$$

wherein f is the factor of internal field F; χ is the polarization direction of electric vector of the absorbed light; $B_\chi = \frac{\Delta\bar{\alpha}}{2hc}$ and $C_\chi = \frac{|\Delta\mu|^2}{6h^2c^2}$ when the angle between $\boldsymbol{F}$ and $\boldsymbol{\chi}$ is 54.7 degrees.

As seen in Supplementary Figure 5, the EA spectrum red QDs can be fitted using the 1st derivate of linear absorption curve. Marked by the dash-lines (in Supplementary Figure 5a, b, c), the extracted Gaussian peaks, which cumulatively fit the measured linear absorption curve, are centered (maximum value) at the energy (or wavelength) where the EA curve intercepts with the y=0 axis. That means the 1st derivative of the first three excitonic peaks are responsible for the first three oscillation features in the EA spectrum. Since our discussion only covers the feature due to 1st excitonic level and its field dependence, a complete fitting of spectrum is out of the scope of the manuscript.

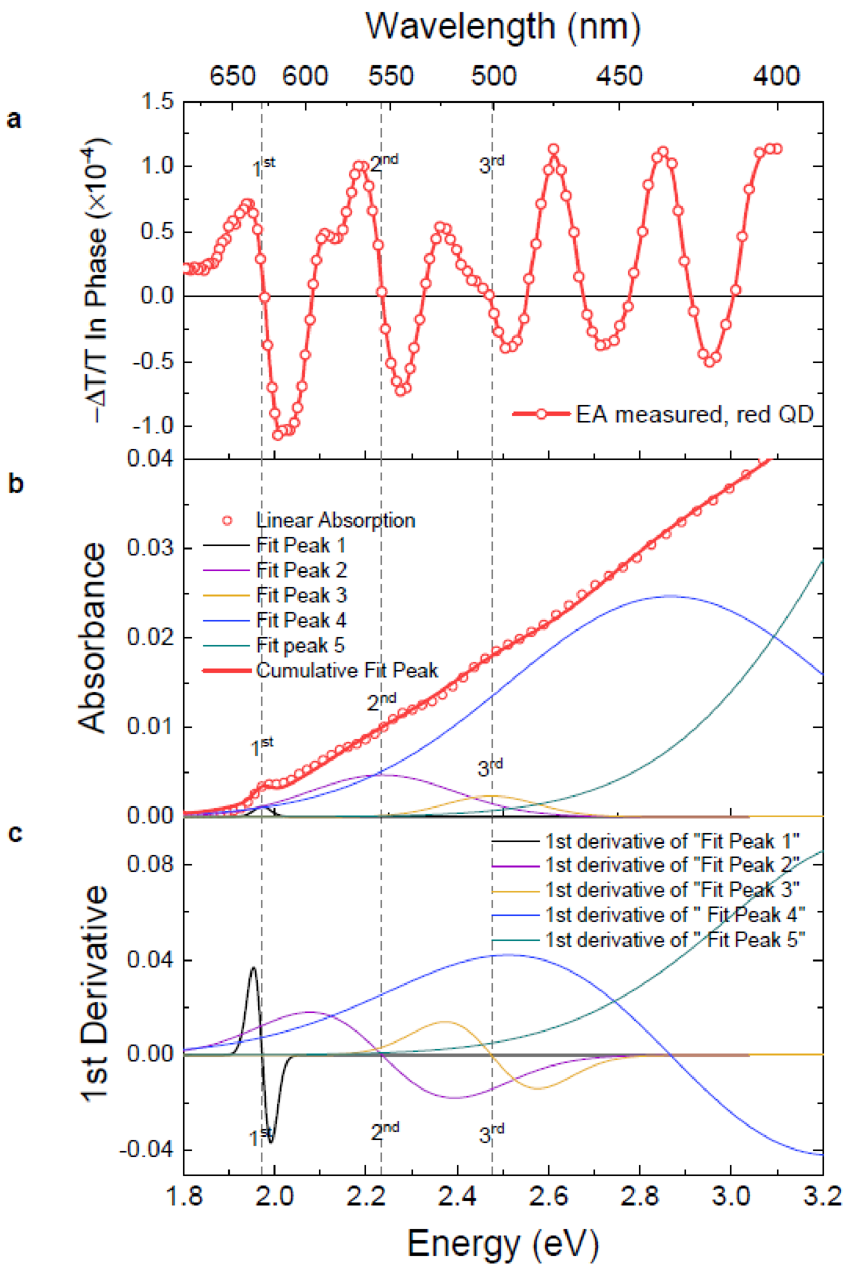

Supplementary Figure 5. Peak assignment. a The electro-absorption spectrum of red quantum dots (QDs). **b** The linear absorption spectrum of red QDs with Gaussian fitting. **c** The 1st derivative of each Gaussian fit peak.

Supplementary Note 5. Energy level diagram

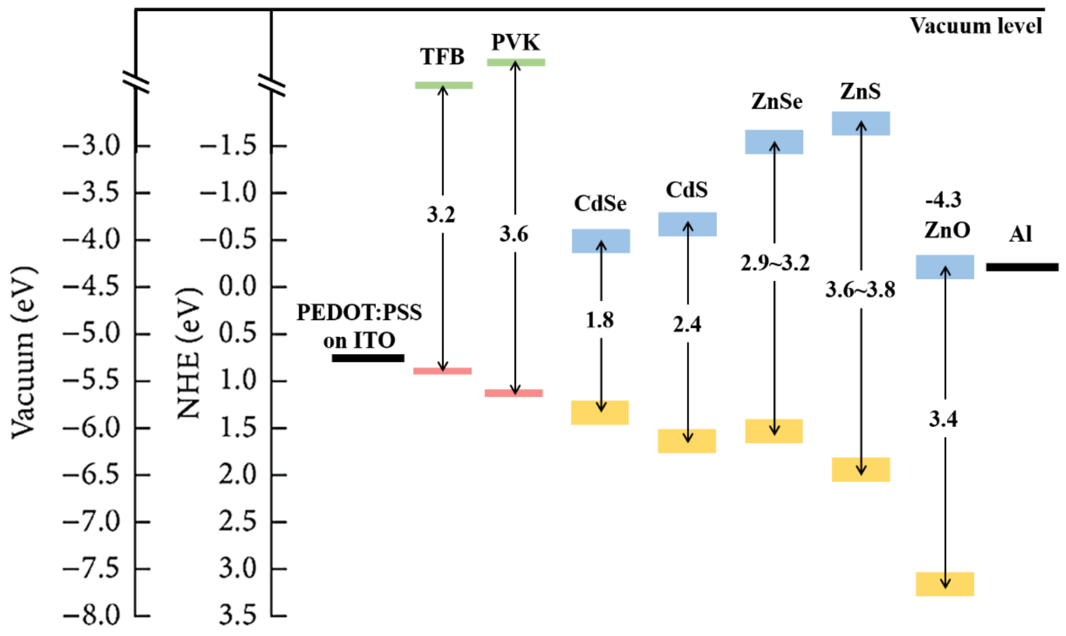

Supplementary Figure 6. **Energy level diagram.** The energy levels of electrodes, charge transporting materials, and II-VI materials used to compose gradient-alloyed QDs ($Cd_{1-x}Zn_xSe_{1-y}S_y$ and $Cd_{1-x}Zn_xS$, x, y increases from core to surface). The results of II-VI materials are summarized from literatures wherein pure phased quantum dots or quantum wells were measured.[3-6]

Profiling the electronic structures of gradiant-alloyed nanomaterials (i.e. $Cd_{1-x}Zn_xSe_{1-y}S_y$ or $Cd_{1-x}Zn_xS$, x, y increases from core to surface) is very challenging. As mentioned in the following paragraph, a complete energy-level diagram of QLED involving gradiant-alloyed QDs has yet to be accurately characterized by (inverse) photoelectron spectroscopy (UPS/ IPES). Although Supplementary Figure 6 cannot accurately reflect the energy alignment in real devcies, it still serves as a reference for materials selection. For example, a thick shell of ZnS has negative impact on the carrier injection.

In our recent publication (W. Cao, *et al*. *Nature Communications*, vol. 9, 2018.), we showed that UPS can resolve the "effective" VBM. In an gradiant-alloyed QD, the "effective" VBM is determined by the electronic structure of pure phase materials, and meanwhile it is sensitive to the detection depth as well as the materials composition which changes continuously from the core to the surface. Therefore, the result helps to demonstarte the change of hole-injection barrier due to surface modification (W. Cao, *et al*.). For "effective" CBM, a direct measruement (e.g. IPES) is even more challenging. Moreover, it is incorrect to calculate the "effective" CBM using the "effective" VBM and optical band gap because the difference between "effective" CBM and "effective" VBM does not necessarily equal to the transport gap, and also because the difference between transport gap and optical gap cannot be determined.

Supplementary Note 6. Capacitance-Voltage characteristics of blue QLED with PVK as HTL

Blue devices with PVK as HTLs are known for high efficiency but very short lifetime. The corresponding *C*-*V* characteristics are shown in Supplementary Figure 7. Due to the slow but balanced charge injection, the capacitance-rise at forward bias is not significant as compared to the case of TFB HTL. Due to the very fast degradation of PVK, devices typically fail when capacitance increases by ~ 30%.

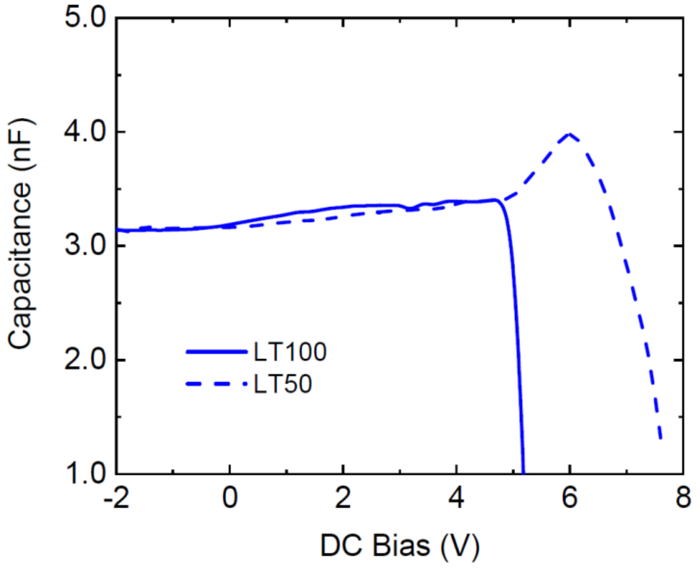

Supplementary Figure 7. **Capacitance-voltage characteristics.** The blue sample with PVK as the HTL is measured before and after the lifetime test.

Suepplementary Table 1. Data summary showing the effect of charge modulation only occurs to the samples with blue QDs and ZnO

Sample structure[a)]	Figure	QD	ZnO	Linear bias dependence of in-phase signal	|Quadrature signal|[b)]
QD only	4(a)	Red	no	Yes	2.0×10^{-6}
QD only	4(c)	Blue	no	Yes	1.2×10^{-6}
QD-ETL	4(b), 5(a)	Red	ETL	Yes	9.0×10^{-6}
QD-ETL	4(d), 5(b)	**Blue**	**ETL**	**No**	$\mathbf{1.0\times10^{-4}}$
HTL-QD-ETL	2(a), S2, 5(c)	Red	ETL	Yes	4.6×10^{-6}
HTL-QD-ETL	2(b), S4(a), S4(c) 5(d)	**Blue**	**ETL**	**No**	$\mathbf{4.0{\sim}6.0\times10^{-5}}$

[a)]The structure also include a pair of electrodes; [b)] V_{dc} = -1V and V_{ac} =0.1 V, the red samples were probed at the wavelength of 570 nm; the blue ones were probed at 455 nm.

Supplementary References

1 Bublitz, G. U. & Boxer, S. G. STARK SPECTROSCOPY: Applications in Chemistry, Biology, and Materials Science. *Annu. Rev. Phys. Chem.* **48**, 213-242 (1997).

2 Awasthi, K., Iimori, T. & Ohta, N. Integral Method Analysis of Electroabsorption Spectra and Its Application to Quantum Dots of PbSe. *J. Phys. Chem. C* **118**, 18170-18176 (2014).

3 Dinger, A., Petillon, S., Grün, M., Hetterich, M. & Klingshirn, C. Conduction band offset of the CdS/ZnSe heterostructure. *Semicond. Sci. Tech.* **14**, 595-598 (1999).

4 Wu, W., Changzhong, J. & Roy, V. A. L. Recent progress in magnetic iron oxide–semiconductor composite nanomaterials as promising photocatalysts. *Nanoscale* **7**, 38-58 (2015).

5 Kumar, P. & Singh, K. Wurtzite ZnSe quantum dots: synthesis, characterization and PL properties. *J. Optoelectron. Biomed. Mater* **1**, 59-69 (2009).

6 Walter, J. P. & Cohen, M. L. Calculation of the reflectivity, modulated reflectivity, and band structure of GaAs, GaP, ZnSe, and ZnS. *Phys. Rev.* **183**, 763 (1969).
